# Free Fatty Acid Receptor 1 Signaling Contributes to Migration, MMP-9 Activity, and Expression of IL-8 Induced by Linoleic Acid in HaCaT Cells

**DOI:** 10.3389/fphar.2020.00595

**Published:** 2020-05-05

**Authors:** Carolina Manosalva, Pablo Alarcón, Karina González, Jorge Soto, Karin Igor, Fernanda Peña, Gustavo Medina, Rafael A. Burgos, María A. Hidalgo

**Affiliations:** ^1^Faculty of Science, Institute of Pharmacy, Universidad Austral de Chile, Valdivia, Chile; ^2^Laboratory of Molecular Pharmacology, Faculty of Veterinary Science, Institute of Pharmacology, Universidad Austral de Chile, Valdivia, Chile; ^3^Department of Diagnostic Processes and Evaluation, Faculty of Health Sciences, Universidad Católica de Temuco, Temuco, Chile

**Keywords:** linoleic acid, free fatty acids receptor 1, keratinocytes (HaCaT), neutrophils, migration, matrix metalloproteinase-9, interleukin-8

## Abstract

Keratinocytes and neutrophils are the main cellular components in wound healing during re-epithelization and inflammation. Free fatty acids such as linoleic acid (LA) present beneficial properties for wound healing by modulating the inflammatory response. LA is a natural ligand of free fatty acids receptor 1 (FFA1), a G protein-coupled receptor (GPCR), able to modulate inflammatory process; however, the role of FFA1 in keratinocytes and wound healing remains poorly understood. In this study, we investigated the role of FFA1 signaling in migration, matrix metalloproteinase-9 (MMP-9) activity, and IL-8 expression induced by LA in keratinocytes. We confirmed that HaCaT cells, a human keratinocyte cell line, expresses the FFA1 receptor and GW1100, a selective antagonist of FFA1, decreased LA-induced migration of HaCaT cells. Also, GW9508, a synthetic agonist of FFA1, increased migration of these cells. Furthermore, ERK1/2 and p38 MAPK inhibitors abolished the LA-induced increase in cell migration. Besides, HaCaT cells stimulated with LA or GW9508 increased the activity of MMP-9 and the expression of IL-8. GW1100 partially inhibited both responses. We further evaluated the effects of HaCaT cells conditioned media stimulated with LA or GW9508 on neutrophil chemotaxis. Conditioned media induced neutrophil chemotaxis. Furthermore, IL-8 secreted by HaCaT cells stimulated with LA or GW9508, contributed to neutrophil chemotaxis. In conclusion, LA increased migration, MMP-9 activity, and expression of IL-8 from HaCaT cells *via* FFA1. Hence, these results showed that the effects induced by LA in keratinocytes can be mediated through FFA1, thus explaining a possible mechanism by which this fatty acid could accelerate wound healing.

## Introduction

Wound healing consists of four phases coordinated temporally and spatially: hemostasis, inflammation, proliferation, and remodeling (Gurtner et al., 2008; Makrantonaki et al., 2017). The inflammatory phase is critical for the prevention of the onset of infections, and neutrophils are the first inflammatory cells that arrive at the damaged tissue (Li et al., 2007). Neutrophils respond to pro-inflammatory cytokines such as interleukin-8 (IL-8), interleukin-1β (IL-1β), and tumor necrosis factor alpha (TNF-α), each of which has been detected in human wound fluid (Landen et al., 2016). Altered recruitment of leukocytes can disrupt wound healing kinetics, delaying the passage from the inflammatory to the proliferative phase, increasing the time required for wound closure (Brubaker et al., 2011; Brubaker and Kovacs, 2013; Ebaid, 2014). Therefore, the inflammatory response influences the following stages of healing. In the proliferative phase, migration of keratinocytes from the wound edge over the provisional matrix is critical to ensure correct closure of the wound (Pastar et al., 2008). Growth factors, such as epidermal growth factor (EGF) and fibroblast growth factor (FGF), accelerate keratinocyte migration and re-epithelialization during wound healing (Yurko et al., 2001; Cheong et al., 2005). Another essential component in keratinocyte migration is secretion of MMP-9, which contributes to basal keratinocyte detachment from the basement membrane (Greenlee et al., 2006; Dufour et al., 2010; Jiang et al., 2014).

Linoleic acid (LA) is an essential ω-6 long-chain polyunsaturated fatty acid found in various vegetable oils such as sunflower oil and soybean oil (Marinho et al., 2013). LA modulates many cellular processes and has been showed to promote wound closure in murine models (Magalhaes et al., 2008; Pereira et al., 2008). Also, LA has been associated with increased numbers of neutrophils in the injured area and acceleration of the early inflammatory phase (Rodrigues et al., 2012). However, the mechanism of action of LA in these responses has not been characterized.

LA is a natural ligand of free fatty acids receptor 1 (FFA1), a G protein-coupled receptor (GPCR) that activates the MAPK signaling pathway (Itoh et al., 2003). Activation of this pathway involves activation of ERK1/2 and p38, which are involved in various cellular functions, such as migration and cell proliferation (Itoh et al., 2003; He et al., 2012). FFA1 is expressed in HaCaT and Normal Human Epidermal Keratinocytes (NHEK) cells (Fujita et al., 2011), and treatment with a synthetic FFA1 agonist, GW9508, decreased expression of CCL17 and CCL5 in HaCaT cells stimulated with TNFα + IFNγ (Fujita et al., 2011). These findings demonstrated that FFA1 regulates the allergic inflammation in the skin, however, the role of LA and FFA1 in keratinocytes and wound healing has not been assessed yet.

In the present study, we confirmed the expression of FFA1 in HaCaT cells and analyzed their contribution in the migration, activity of MMP-9 and expression of IL-8 induced by linoleic acid. Additionally, we evaluated the conditioned media of HaCaT cells stimulated with FFA1 agonists in neutrophil chemotaxis.

## Materials and Methods

### Reagents and Antibodies

Linoleic acid and GW9508 were purchased from Sigma-Aldrich (St, Louis, MO, USA). EGF was obtained from ThermoFisher (Frederick, MD, USA). Phospho-ERK, phospho-p38, total ERK, total p38, and GAPDH antibodies were purchased from Santa Cruz Biotechnology (Santa Cruz, CA, USA). FFA1 antibody was obtained from Abcam (ab75197, USA). Inhibitors UO126 and SB203580 were obtained from Promega (Madison, WI, USA).

### Cell Culture

The HaCaT cell line (Boukamp et al., 1988) was kindly donated by Dr. Miguel Concha at the Department of Pathology, Faculty of Medicine, Universidad Austral de Chile, Valdivia, Chile. Cells were cultured in Dulbecco’s Modiﬁed Eagle’s Medium (DMEM; Mediatech, Manassas, VA, USA) supplemented with 10% fetal bovine serum (FBS; Gibco, Grand Island, NY, USA), and a mix of penicillin and streptomycin (Mediatech, Manassas, VA, USA) at 37°C in a humidified incubator containing 5% CO_2_.

### Cell Viability Assays

Cells were seeded into 96 well plates at 1 × 10^4^ cells/100 μl and incubated at 37°C for 24 h. Concentrations of 50 µM and 100 µM LA were added to each well for 48 h. Cell viability was determined using cell counting kit-8 (CCK-8) (Sigma-Aldrich, St. Louis, MO, USA). Brieﬂy, 10 µl of CCK-8, that contained WST-8 (2-(2-methoxy-4-nitrophenyl)-3-(4-nitrophenyl)-5-(2,4-disulfophenyl)-2H-tetrazolium, monosodium salt) which is transformed in formazan dye and is proportionality the living cells, was added to the cells and incubated for 2 h at 37°C in 5% CO_2_. The developed color was measured at 450 nm using a plate reader (Stat Fax ^®^ 2100, Palm City, FL, USA). The stock solution of linoleic acid (100%) was stored at −20°C, hermetically sealed, and protected from light. To perform the experiments LA was dissolved in DMSO (0.1%V/V) and was used immediately and protected from light to retard the oxidation process.

### Scratch Wound Assay

An *in vitro* wound-healing model of cultured HaCaT cells was used according to previous studies (Nasca et al., 1999). Briefly, HaCaT cells suspended in DMEM were seeded in 12 well plates at a density of 2 × 10^5^ cells/ml per well and then incubated at 37°C until the cells reached 100% confluence. Mitomycin-C was added at a final concentration of 10 µg/ml, and the cells were incubated for an additional 2 h to inhibit cell proliferation. A sterile plastic 20 µl pipette tip was used to scratch the confluent cell monolayer evenly in each well to generate a cell-free zone. After washing away the floating cells with phosphate-buffered saline (PBS), new media containing 50 µM or 100 µM LA and 10 µM or 50 µM GW9508 was added to each well. These concentrations have been used by other authors to evaluate effects on cells constitutively expressing FFA1 receptor (Kotarsky et al., 2003; Zhou et al., 2012; Wauquier et al., 2013; Li et al., 2016; Puebla et al., 2016; Matoba et al., 2018).

In another set of experiment, HaCaT cells were pre-incubated with 10 μM U0126 or 10 μM SB203580 for 30 min or 10 μM GW1100 for 15 min, and then stimulated with 50 μM LA. Dimethyl sulfoxide (0.1% DMSO) was used as the control. Cell migration into the wound space was examined at 0 and 24 h after wounding using an inverted microscope equipped with a digital camera. Images were visualized using ImageJ 1.35s software. Wound closure was determined as the difference between wound areas at times 0 and 24 h. At least five scratched regions were randomly chosen for each sample, and the averages were calculated.

### Transwell Migration Assay

HaCaT cells were seeded (1×10^5^ cells/well) into 24-well plates in DMEM containing 1% FBS onto a microporous membrane (8.0 µm) in the upper chamber of a Transwell (Corning, Kennebunk, ME, USA). Twenty-four hours after treatment with LA or GW9508, the remaining cells in the upper chamber were gently removed using a cotton swab. Cells that had migrated through the membrane to the lower chamber were fixed in 4% paraformaldehyde, stained with 0.5% crystal violet, and washed with PBS. Migration was determined by counting the cells on the lower surface of the filter using phase-contrast microscopy and using ImageJ 1.35s software to count cells. Cells were counted in at least five randomly chosen fields for each sample, and the averages were calculated. In addition, we quantify the percentage of stained area, in those cases where a high confluence of cells made accounting difficult. The percentage of stained area was obtained using the ImageJ 1.35s software.

### Immunoblot

To measure MAPK phosphorylation, confluent HaCaT cells were treated with 50 μM LA or 50 μM GW9508 across a range of times (0, 5, 15, 30, and 60 min). In another set of experiments, HaCaT cells were pre-incubated with 10 μM GW1100 for 15 min at 37°C, then stimulated with 50 μM LA for 15 min. After stimulation, the cells were lysed with lysis buffer (50 mM Tris–HCl, pH 7.4, 150 mM NaCl, 1 mM EDTA, 1 mM EGTA, 10 μg/ml aprotinin, 10 μg/ml leupeptin, 5 mM phenylmethylsulfonyl fluoride (PMSF), and 1 mM DTT) on ice. Insoluble debris was removed by centrifugation at 18,000*g* for 20 min at 4°C, and protein content was determined using Bradford reagent (Sigma-Aldrich, Saint Louis, MO, USA). Fifty micrograms of protein were resolved on 10% SDS–PAGE gels and transferred to PVDF membranes. Membranes were blocked with 5% skim milk in TBS/T (20 mM Tris–HCl, pH 7.6, 137 mM NaCl, and 0.05% Tween 20) and incubated with antibodies against pERK1/2 and phospho-p38 (Santa Cruz, CA, USA). The primary antibodies were stripped, and each membrane was incubated with anti-ERK1/2 or anti-p38 antibodies (Santa Cruz, CA, USA). For determination of FFA1, membranes were incubated with an anti-human FFA1 antibody (Abcam, USA) and an anti-GAPDH antibody (Santa Cruz, CA, USA), which was used as a loading control. Membranes were then incubated with an HRP-conjugated anti-rabbit secondary antibody (Santa Cruz, CA, USA) and bands were visualized using an enhanced chemiluminescence system (UVP ChemiD-itTS2., CA, USA). Band density was measured using ImageJ 1.35s software.

### Quantitative Real-Time PCR

Total RNA was extracted from HaCaT cells using RNA Kit I (Omega Bio-Tek Inc, Norcross, GA, USA) according to the manufacturer’s protocol. RNA was treated with DNAse to ensure removal of genomic DNA. Equal amounts of RNA (1 μg) were reverse transcribed using M-MLV Reverse Transcriptase (Promega, Madison, WI, USA). Real-time PCR was performed using Brilliant II SYBRGreen qPCR (Stratagen) using the following primers: FFA1 forward 5′-ctggtctacgccctgaacct-3′ and reverse 5′-gagcctccaacccaaagacc-3′; FFAR4 forward 5′-gaaatttcgatttgcacact-3′ and reverse 5′-gtttagggctgaattagcaa-3′ (Fujita et al., 2011); hIL-8 forward 5′-agacagcagagcacacaagc-3′ and reverse 5′-atggttccttccggtggt-3′ (Peric et al., 2008); hGAPDH forward 5′-gtgaaggtcggagtcaacg-3′ and reverse 5′-tgaggtcaatgaaggggtc-3′ (Raingeaud and Pierre, 2005). The expression levels of IL-8 were normalized to the expression of the housekeeping gene GAPDH, then quantified using the 2^−ΔΔCT^ method (Livak and Schmittgen, 2001). The product of FFA1 gene reverse transcription was separated on a 2% agarose gel with SYBR Safe (Invitrogen) for detection.

### Intracellular Ca^+2^ Measurement

HaCaT cells (2 × 10^6^ cells/2 ml) were loaded with 3 μM Fura-2AM fluorescent indicator dye (Sigma-Aldrich, Saint Louis, MO, USA) in Hank’s balanced salt solution (HBSS) for 30 min, washed three times, and incubated for 15 min at room temperature. Cells were incubated with 50 μM LA (Sigma-Aldrich, Saint Louis, MO, USA), 50 μM GW9508 (Cayman Chemical), or vehicle (0.1% DMSO). In another set of experiments, cells were incubated with 10 μM GW1100 (Cayman Chemical, Ann Arbor, MI, USA) for 15 min and then stimulated with 50 μM LA. Cellular fluorescence (Ca^2+^) was measured at 509 nm emission with 340/380 nm dual wavelength excitation using a LS55 spectrofluorimeter (PerkinElmer Life Science). Cuvette temperatures were maintained at 37°C with constant stirring.

### Zymography

Sub-confluent cultures of 1 × 10^5^ cells were cultured in 12-well plates and treated with LA or GW9508 ranging from 0-100 µM for 24 h. In another set of experiments HaCaT cells were pre-incubated with 10 μM GW1100 for 15 min, then stimulated with 50 μM linoleic acid at 37°C for 24 h. To determine MMP-9 activity, we analyzed HaCaT cell supernatants by zymography according to previous studies (Toth et al., 2012). Conditioned medium was collected and centrifuged at 600*g* at 4°C for 10 min and stored at −80°C until MMP-9 measurement. Non-concentrated and non-heated samples, normalized to an equal cell number, were mixed with loading buffer without reducing agent and loaded onto 7.5% acrylamide gels copolymerized with gelatin (1 mg/ml). After electrophoresis at 200 V for 40 min at 4°C, the gels were rinsed twice in 2.5% Triton X-100 to remove the SDS, then incubated with digestion buffer (50 mM Tris/HCl at pH 7.5, 5 mM CaCl_2_ and 200 mM NaCl) overnight at 37°C. The gels were then stained with 0.25% Coomassie Brilliant Blue G250 in 7% acetic acid and 30% methanol. Areas with proteinase activity were visualized as clear bands and analyzed with ImageJ 1.35s software.

### Neutrophil Isolation

Blood samples were obtained from healthy volunteers after obtaining written informed consent. This study was carried out in accordance with the recommendations of “Comisión Nacional de Investigación Científica y Tecnológica.” This study was approved by the Ethics and Bioethics Committee, subcommittee on bioethics in human research, Universidad Austral de Chile.

Blood samples were stored in tubes containing heparin as an anticoagulant (Kuijpers et al., 1991). The blood samples were diluted 1:1 with phosphate-buffered saline (PBS) and placed in discontinuous Percoll gradient (63% at the top and 72% at the bottom of the column), and centrifuged (Kuijpers et al., 1991). The granulocyte-containing pellets were suspended in 50 ml of ice-cold lysis solution and lysis of erythrocytes was performed on ice for 10 to 15 min (Kuijpers et al., 1991). The granulocytes were washed twice, suspended in neutrophil medium, and kept at room temperature (Kuijpers et al., 1991). This method of neutrophil isolation has been reported to yield > 97% pure and > 99% viable cells (Kuijpers et al., 1991). The purity of the obtained neutrophils was analyzed by flow cytometry (BD FACSCanto II; Becton Dickinson) using a forward-scatter versus side-scatter dot plot to determine the relative size and granularity of cells.

### Preparation of Keratinocytes Conditioned Medium

HaCaT cells were cultured in 6-well plates (3 × 10^4^ cells/2 ml) in DMEM with 10% FBS and high in glucose at 37°C and 5% CO_2_ until they reached 80% confluency. The cells were left overnight in medium without FBS. Then, the cells were stimulated with 10, 50, or 100 μM LA and 10, 50, or 100 µM GW9508 for 24 h. In another set of experiments, the cells were pre-incubated with 10 μM GW1100 for 15 min, then stimulated with 50 μM LA for 24 h at 37°C. The keratinocyte conditioned medium (KCM) was harvested and centrifuged at 12,000*g* for 10 min at 4°C. The supernatant was filtered through a 0.22 μM membrane and stored at −80°C until experiments.

### Chemotaxis Assays, Determination of Neutrophils Migration

Chemotaxis assays were performed using transwell filters (3 μm pore size). Neutrophils were suspended in HBSS/Ca^+2^ (2 × 10^5^/100 μl) and placed in the upper compartment of the transwell chamber (Katayama et al., 1994) (**Figure 6A**).

KCM was added to the lower well of the transwell plate at a 1:1 ratio with HBSS/Ca^+2^, to a final volume of 600 μl. The plates were incubated at 37°C for 1 h, the time required for neutrophils to migrate through the polyvinylpyrrolidone-free polycarbonate filters (3μm pore size) which separated the upper and lower compartments (Katayama et al., 1994). Neutrophils that migrated across the filters were counted under a light microscope.

### Statistical Analyses

The results were analyzed by one-way analysis of variance (ANOVA), and Dunnett’s multiples comparison test. A Tukey´s test was used to determinate the significant differences between group. The analysis was done using Graph Pad Prism v5.0 software (Graph Pad software Inc. CA. USA). P < 0.05 was considered significant.

## Results

### LA Increased Human Keratinocytes Migration

**Figure 1:**
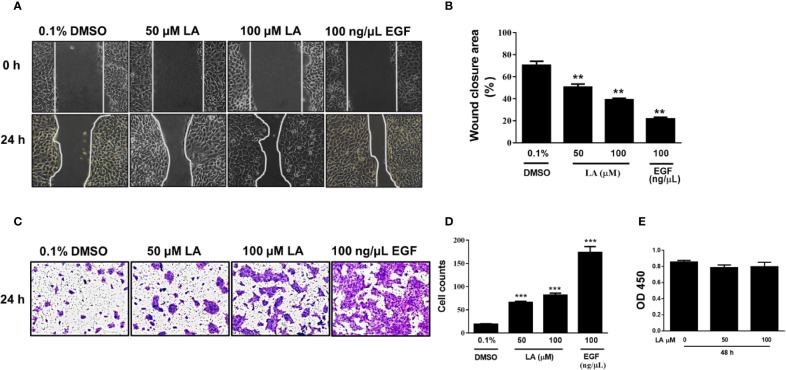
Linoleic acid increased migration of HaCaT cells. HaCaT cells were scratched using a micropipette tip and treated with 0.1% DMSO, 50 µM LA, 100 μM LA, or 10 ng/µl EGF (positive control) for 24 h. **(A)** Representative images of cell-free area size at 0 and 24 h after stimuli are shown. **(B)** Quantification of cell-free area. Wound closure was calculated as the difference between uncovered area at 0 and 24 h after treatment. Data are expressed as mean ± SEM (n = 5). **P < 0.01, compared with control group using Dunnett’s multiple comparison test. **(C)** Migration of HaCaT cells was examined using a transwell migration assay. Representative images are shown. **(D)** The cells that migrated towards the lower side of the membrane were stained with 0.5% crystal violet and then counted in 5 independent fields per condition. Data are expressed as mean ± SEM (n = 5). *** P < 0.001, compared with the control group, using Dunnett’s multiple comparison test. **(E)** After treatment with LA for 48 h, cell viability was detected using a CCK-8 assay. OD values at 450 nm for each group are depicted. Data are expressed as mean ± SEM (n = 5). OD: optical density.

Lateral migration of basal keratinocytes located at the leading edge of the wound is a critical process for re-epithelialization of skin wounds (Woodley et al., 2015). Therefore, we evaluated the effect of LA on the migration of HaCaT cells using the scratch wound-healing assay. Our results showed that HaCaT cells stimulated with 50 µM LA or 100 µM LA for 24 h migrated toward cell-free areas, resulting in significantly better coverage of the wound compared with control (0.1% DMSO) (**Figure 1A**). The wound area percent that remained open after 24 h was 50.61% for 50 μM LA and 39.14% for 100 μM LA. The cell-free area in the control group was 70.4% (**Figure 1B**). To compare the effect of LA on the migration of keratinocytes, we used epidermal growth factor (100 ng/µl EGF), a known stimulator of epithelial migration (Seeger and Paller, 2015). The cells treated with EGF presented a cell-free area of 21.8% after 24 h. To avoid LA-induced proliferation during wound closure, all experiments were performed in the presence of an inhibitor of proliferation, 10 µg/ml mitomycin-C. Therefore, the results demonstrated that LA, at the concentrations studied, promoted migration of HaCaT keratinocytes. LA-induced HaCaT cell migration was further confirmed by transwell migration assay in which the number of cells that migrated through the pores of the membranes were counted after being stimulated with 50 µM LA or 100 μM LA for 24 h (**Figure 1C**). LA increased significantly the number of cells that migrated compared to the control group (**Figure 1D**). A similar result is observed when the percentage of the area stained in the membranes was quantified (**Figure Supplementary 1A**). These results suggested that LA promoted HaCaT cell migration. To evaluate the effect of LA on keratinocyte cytotoxicity, CCK-8 assay was used. We did not observe any changes in cellular viability any concentration of LA (**Figure 1E**), hence these concentrations of LA not were toxic to HaCaT cells.

### Functional Expression of FFA1 Receptor and Role in the Migration of HaCaT Cells

**Figure 2:**
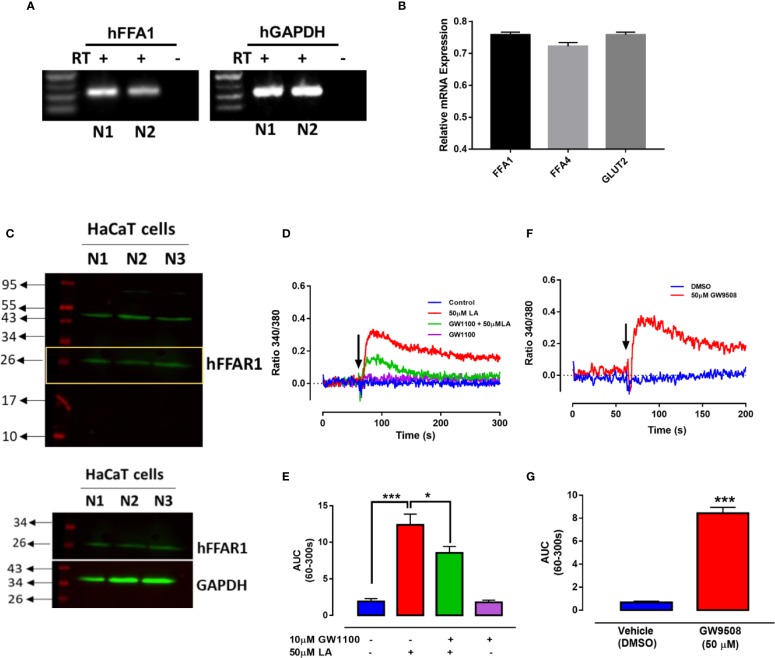
Expression of FFA1 in HaCaT cells. **(A)** RT-PCR was performed using primers specific for human FFA1. –RT, without reverse transcriptase; +RT, with reverse transcriptase of two different samples (N1 and N2). **(B)** Relative mRNA levels of FFAR1, FFAR4 and GLUT2 were quantified by qRT-PCR from HaCaT cells normalized with GAPDH (housekeeping). **(C)** Western blot of FFA1 from totals protein of three different batches of HaCaT cells (N1, N2, and N3). **(D–G)** Intracellular calcium in HaCaT cells was measured by spectrofluorimetry using the fluorescent probe Fura2-AM. **(D)** A representative register of 50μM LA in presence or absence of GW1100 and **(E)** area under the curve (AUC) between 60–300 s are shown. **(F)** A representative register of 50μM GW9508 and **(G)** area under the curve (AUC) between 60–300 s. Arrows represent the time of solvent (control) or ligand addition. Mean ± SEM of 4 independent experiments. * p < 0.05, *** p < 0.001 compared with the control or 50 μM LA.

To confirm the presence of the FFA1 receptor in HaCaT cells, we first obtained total RNA from HaCaT cells for determination of FFA1 mRNA using specific primers (Fujita et al., 2011). We obtained a product of 316 bp (**Figure 2A**), coinciding with the results described by Fujita et al. (2011). FFA1 expression levels were compared with the expression level of FFA4 and GLUT2, which have been previously determined in HaCaT cells (Harel et al., 2002; Fujita et al., 2011) (**Figure 2B**). Western blot was performed using 3 different HaCaT cell clones. A band with an approximate size of 26 kDa was obtained, similar to the size observed in the antibody datasheet, however, also a predicted size of 31 kDa is reported. The size obtained for FFA1 protein in HaCaT cells is similar to that reported in other cells, that constitutively expressing this receptor (Nishinaka et al., 2014; Mizuta et al., 2015; Puebla et al., 2016) (**Figure 2C**). These differences may be due to splicing variants or posttranslational modifications that occur in a protein, such as phosphorylation and glycosylation. Activation of FFA1 by its ligands has been shown to produce an increase in intracellular calcium in different cells (Yonezawa et al., 2004; Zhao et al., 2013; Mizuta et al., 2015). Thus, to determine the functionality of the FFA1 receptor in HaCaT cells, we evaluate intracellular calcium mobilization in cells loaded with the Fura 2-AM probe. The curve representing 50 μM LA-induced increase in intracellular calcium showed a rapid increase, followed by a slight decrease. The analysis of the area under the curve (AUC) between 60 and 300 s (AUC _(60–300_ _s)_) revealed a significant increase in intracellular calcium induced by 50 μM LA and a significant decrease in presence of GW1100 (**Figures 2D, E**). In addition, 50 μM GW9508 increased calcium mobilization confirming receptor functionality (**Figures 2F, G**).

**Figure 3:**
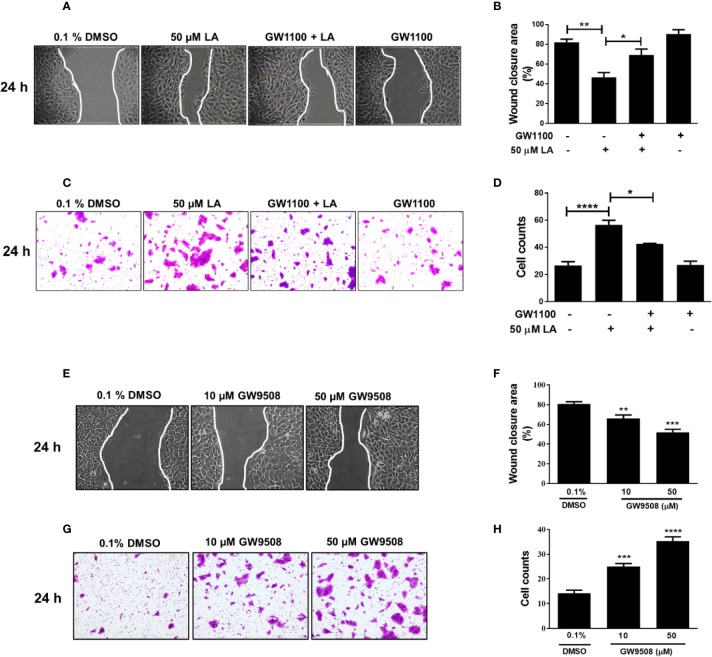
FFA1 contributed to migration of HaCaT cells. **(A**–**D)** Effect of GW1100 on migration of HaCaT cells induced by LA. Bars indicate the mean ± SEM of three independent experiments. *P <0.05, **P < 0.01, ****P < 0.0001; compared with control or 50 µM LA, using Tukey’s multiple comparison test. **(E)** Representative pictures of experiments after 24 h of treatment are shown. **(F)** Quantification of cell-free area. Wound closure was calculated as the difference between uncovered area at 0 and 24 h after treatment. Data are expressed as mean ± SEM (n = 5). **P < 0.01 and ***P < 0.001 compared with control group using Dunnett’s multiple comparison test. **(G)** Migration of HaCaT cells was examined using a transwell migration assay. Representative pictures from five independent experiments are shown. **(H)** Bars indicate the mean ± SEM of number of migrating cells from five independent experiment. *** *P* < 0.001 and **** *P* < 0.0001 compared with the control group, using Dunnett’s multiple comparison test.

Next, to investigate the role of FFA1 in LA-induced migration of HaCaT cells, we evaluated the effect of GW1100, a selective antagonist of FFA1. We observed that in the presence of GW1100, LA significantly increased the wound closure area (**Figures 3A, B**), which was consistent with the significant decrease in the number of cells that migrated in the transwell assay (**Figures 3C, D**; **Figure Supplementary 1B**). These results suggest that LA can mediate the migration of HaCaT cells through FFA1 receptor. Additionally, the synthetic FFA1 agonist, GW9508, significantly decreased the cell-free area compared to the control. Cells exposed to 10 μM or 50 μM GW9508 had cell free areas of 66.2%, and 51.9% respectively, compared with the control which had an open wound area of 86.7% (**Figures 3E, F**). On the other hand, 10 and 50 µM GW9508 significantly increased migration of the cells compared with the control in the transwell migration assay (**Figures 3G, H**; **Figure Supplementary 1C**).

### Activation of ERK1/2 and p38 MAPK Is Required for Migration Induced by Linoleic Acid in HaCaT Cells

**Figure 4:**
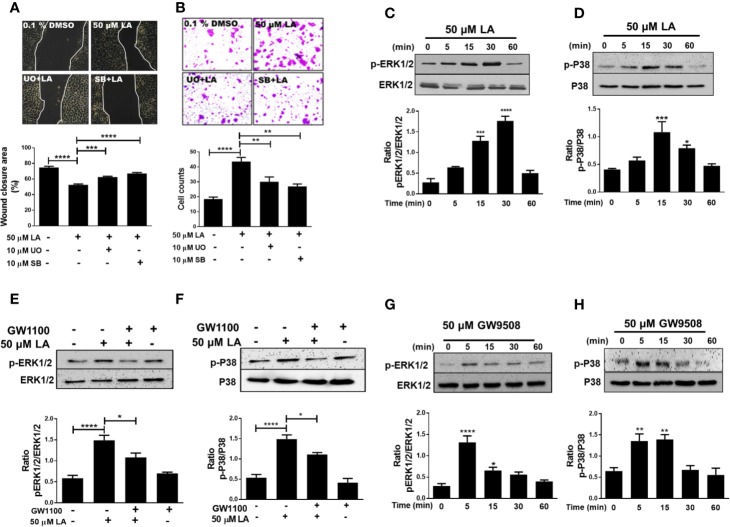
Involvement of ERK1/2 and p38 MAPK in linoleic acid induced HaCaT cell migration. **(A)** HaCaT cells were pre-incubated with 10 μM U0126 or 10 μM SB203580 for 30 min and then stimulated with 50 μM LA. Representative pictures of scratch wound assays from five independent experiments are shown. Wound closure rates, recorded 24 h after scratch wounding of HaCaT monolayers, were calculated as the difference between uncovered area at 0 and 24 h. Bars represent mean ± SEM of five independent experiments. ***P < 0.001 and ****P < 0.0001, compared with control or linoleic acid treatment, using Tukey’s multiple comparison test. **(B)** Representative pictures from five independent experiments of transwell migration assay are shown. Bars indicate the mean ± SEM of number of migrating cells from five independent experiments. **P < 0.01 and ****P < 0.0001 compared with control or linoleic acid-treated cells, using Tukey’s multiple comparison test. **(C, D)** HaCaT cells were treated with 50 μM LA across a range of time points (0, 5, 15, 30, and 60 min). Total protein was analyzed by SDS/PAGE and immunoblotting using speciﬁc antibodies against the phosphorylated forms of ERK1/2 and p38. Total p38 or ERK1/2 were also evaluated by western blot for comparison. The images of one representative experiment are shown. The densitometry ratios of pERK/ERK and p-p38/p38 are shown. Each bar represents mean ± SEM of five independent experiments. *P < 0.05, ***P < 0.001, and ****P < 0.0001 compared with the control, using Dunnett’s multiple comparison test. **(E, F)** HaCaT were treated with 10 µM GW1100 for 15 min and then stimulated with 50 µM LA. Totals protein was analyzed by immunoblotting for p-ERK1/2 and p-P38. The densitometry ratios of pERK/ERK and pP38/P38 are shown. Bars indicate the mean ± SEM of five independent experiments. *P < 0.05 and ****P < 0.0001 compared with control or linoleic acid-treated cells, using Tukey’s multiple comparison test. **(G, H)** HaCaT cells were treated with 50 µM GW9508 across a range of time points (0, 5, 15, 30, and 60 min). Total protein was analyzed by SDS/PAGE and immunoblotting using speciﬁc antibodies against the phosphorylated forms of ERK1/2 and p38. Total p38 or ERK1/2 were also evaluated by western blot for comparison. The images of one representative experiment are shown. The densitometry ratios of pERK/ERK and p-p38/p38 are shown. Each bar represents mean ± SEM of five independent experiments. *P < 0.05, **P <0.01, and ****P < 0.0001 compared with the control, using Dunnett’s multiple comparison test.

ERK1/2 and p38 MAPK phosphorylation are associated with regulation of cell migration (Huang et al., 2004). We examined if U0126 and SB 203580, which are inhibitors of MEK and p38, respectively, could reverse LA-induced migration of HaCaT cells. The results showed that U0126 and SB203580 inhibited 50 µM LA-induced HaCaT cell migration (**Figure 4A**). In addition, both inhibitors decreased the number of cells that migrated toward LA in the transwell migration assay (**Figure 4B**; **Figure Supplementary 1D**), suggesting that migration induced by LA was dependent of the activation of ERK1/2 and p38. Thus, we evaluated whether LA increased phosphorylation of ERK1/2 and p38 MAPK in HaCaT cells. We found that 50 µM LA treatment increased phosphorylation of ERK1/2 from 5 min after stimulation (**Figure 4C**), and ERK1/2 phosphorylation peaked at 30 min after stimulation. Besides, LA increased phosphorylation of p38 after 15 min of incubation (**Figure 4D**).

Recent studies showed that activation of the FFA1 receptor by long-chain fatty acids increased phosphorylation of ERK1/2 and p38 (Mena et al., 2016; Qian et al., 2017), which is consistent with the decrease in LA-induced phosphorylation in the presence of GW1100, observed in our study (**Figures 4E, F**). Also, cells stimulated with 50 μM GW9508 increased phosphorylation of ERK1/2 and p38 MAPK, which peaked at 5 min and then decreased to basal levels (**Figures 4G, H**). Hence, our results showed that LA modulated the MAPK signaling pathway in HaCaT cells by increasing the phosphorylation of ERK1/2 and p38 through FFA1.

### Involvement of FFA1 Receptor in MMP-9 Activity and IL-8 Expression in HaCaT Cells

**Figure 5:**
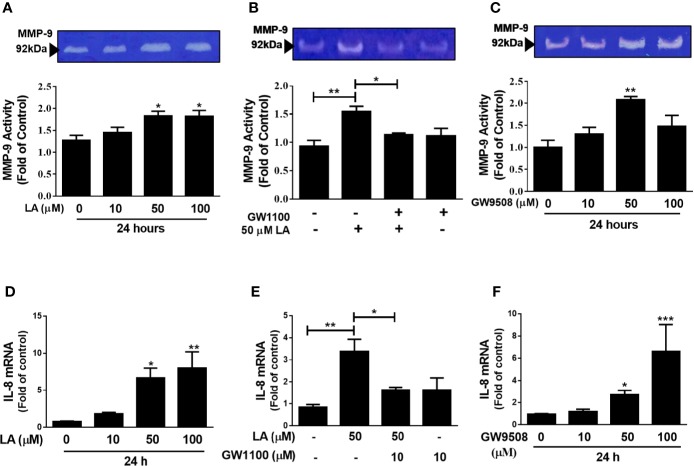
Involvement of FFA1 in modulation of MMP-9 activity and IL-8 expression in HaCaT keratinocytes. **(A)** HaCaT cells were incubated with 10 µM, 50 µM, or 100 μM LA for 24 h and MMP-9 activity was analyzed in the supernatant of cultured HaCaT cells by zymography. Each bar represents mean ± SEM, n = 5, *P < 0.05, compared with the untreated control, using Dunnett’s multiple comparison test. **(B)** HaCaT cells were treated with 10 µM GW1100 for 15 min and then stimulated with 50 µM LA for 24 h and MMP-9 activation was analyzed. The data are represented as the mean ± SEM of five independent experiments. **P < 0.01 compared with control and *P < 0.05 compared with linoleic acid-treated cells, using Tukey’s multiple comparison test. **(C)** HaCaT cells were incubated with 10 µM, 50 µM, or 100 μM GW9508 for 24 h and MMP-9 activity was analyzed in the supernatant of cultured HaCaT cells by zymography. Each bar represents mean ± SEM, n = 5, **P < 0.01 compared with the untreated control, using Dunnett’s multiple comparison test. mRNA expression of IL-8 was analyzed by qRT-PCR. HaCaT cells were incubated with **(D)** LA **(E)** 10 µM GW1100 for 15 min and then stimulated with 50 µM LA for 24 h and **(F)** GW9508. The data are represented as the mean ± SEM of five experiments. *P < 0.05, **P <0.01 compared with control, using Dunnett’s multiple comparison test. *P < 0.05, **P < 0.01, and ***P < 0.001 compared with control or linoleic acid-treated cells using Tukey’s multiple comparison test.

MMP-9 has been shown to contribute to keratinocyte migration during wound repair (Raja et al., 2007). In addition, IL-8 has a direct effect on migration of keratinocytes and is an important chemoattractant of pro-inflammatory cells, which are essential for correct wound closure (Jiang et al., 2012; Rees et al., 2015). In this study, we evaluated the effect of LA on MMP-9 activity by zymography and IL-8 expression by qRT-PCR. Our results showed a significant increase in MMP-9 activity in the supernatants of cultured HaCaT cells up to 24 h after stimulation with 50 μM or 100 μM LA (**Figure 5A**). Additionally, pre-treatment of the cells with GW1100 decreased LA-induced MMP-9 activity (**Figure 5B**), which suggested that FFA1 can regulate the activity of MMP-9, a key enzyme involved in cell migration, in keratinocytes. We also showed that 50 μM GW9508 increased MMP-9 activity at 24 h after stimulation (**Figure 5C**).

Similarly, treatment with 50 µM LA or GW9508 resulted in increased IL-8 expression and the effect of LA was reduced by pre-treatment with the FFA1 receptor antagonist (**Figures 5D–F**). These results suggested that agonists of the FFA1 receptor modulate the activity of MMP-9 and the expression of IL-8 in HaCaT keratinocytes.

### Conditioned Medium of HaCaT Cells Stimulated With FFA1 Agonists Increased Neutrophil Migration Through of IL-8

**Figure 6:**
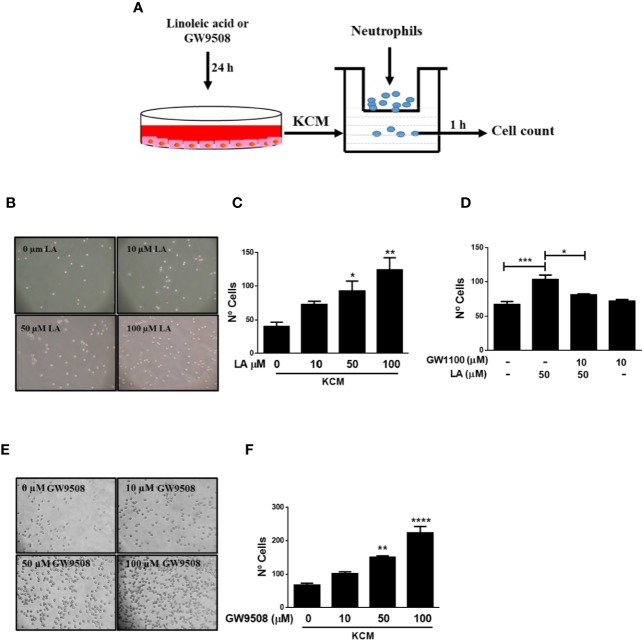
Conditioned medium of HaCaT cells stimulated with FFA1 agonists increased chemotaxis of neutrophils. **(A)** Neutrophils were isolated from the blood of healthy donors and analyzed by flow cytometry prior to use in a transwell assay using the conditioned medium of HaCaT cells as a chemoattractant. **(B, C)** Neutrophil chemotaxis was analyzed using conditioned media from HaCaT cells treated with different concentrations of LA (10–100 μM) for 24 h. The data are represented as the number of neutrophils that migrated to the conditioned medium. The data are the mean ± SEM of five blood donors. *P < 0.05 and **P < 0.01, compared with the control, using Dunnett’s multiple comparison test. **(D)** Conditioned media of HaCaT treated with 10 µM GW1100 for 15 min and then stimulated with 50 µM LA for 24 h was used to evaluate neutrophil chemotaxis. The data are the mean ± SEM of five blood donors. *P < 0.05 and ***P < 0.001, compared with the control or linoleic acid-treated cells using Tukey’s multiple comparison test. **(E, F)** Neutrophil chemotaxis was analyzed using conditioned media from HaCaT cells treated with different concentrations of GW9508 (10–100 μM) for 24 h. The data are represented as the number of neutrophils that migrated to the conditioned medium. The data are the mean ± SEM of five blood donors. **P < 0.01, and ****P < 0.0001, compared with the control, using Dunnett’s multiple comparison test.

A previous study showed that long-chain fatty acids, such as LA and oleic acid (OA) accelerated the early inflammatory phase and thus promoted wound closure in rats (Rodrigues et al., 2012). Moreover, Pereira et al. (2008) showed that topical administration of these fatty acids to wounds increased the number of neutrophils in the injured area (Pereira et al., 2008). These authors also demonstrated that OA and LA activated neutrophils, which promoted a pro-inflammatory state and contributed to wound healing (Pereira et al., 2008). We hypothesized that the effects of LA on neutrophil migration occur *via* indirect stimulation. To evaluate this hypothesis, we obtained conditioned media from HaCaT cells treated with LA for 24 h. Using a transwell migration assay, we observed that the conditioned media of HaCaT cells stimulated with 50 and 100 μM linoleic acid (KCM-LA) significantly increased migration of neutrophils into the media (**Figures 6B, C**). To evaluate the role of FFA1 in neutrophil chemotaxis induced by KCM-LA, HaCaT cells were pretreated with GW1100, then stimulated with 50 µM LA to obtain conditioned media. The number of neutrophils that migrated toward the GW1100/LA-induced conditioned media was lower than that induced by the conditioned media of HaCaT cells stimulated only with LA (**Figure 6D**). These findings suggested that LA stimulated HaCaT cells to secrete factors soluble in the media, possibly through FFA1 signaling, which mediated recruitment of neutrophils. In addition, conditioned media of HaCaT cells treated with GW9508 (KCM-GW) increased neutrophil chemotaxis toward the media (**Figures 6E, F**).

**Figure 7:**
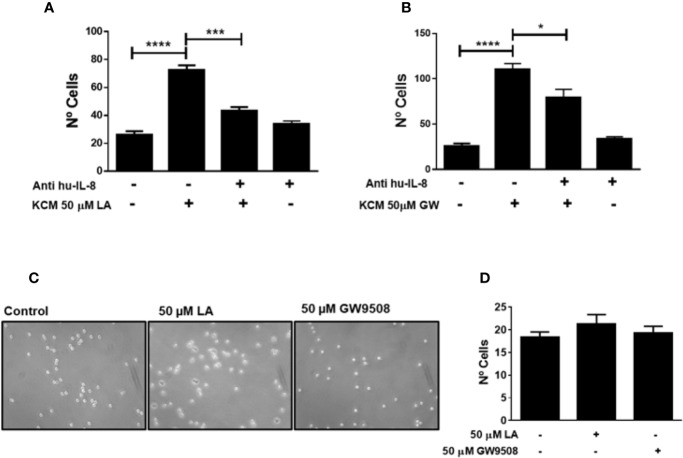
Neutrophil chemotaxis induced by KCM-LA or KCM-GW is dependent on IL-8. **(A, (B)** The conditioned medium of HaCaT cells treated with 50 μM LA or 50 µM GW9508 was pre-treated for 10 min with anti-human IL-8 (1 μg/ml). The data are the mean ± SEM of 3 experiments. *P < 0.05, *** P <0.001 and ****P < 0.0001, using Tukey’s multiple comparison test. **(C, D)** Neutrophil chemotaxis was analyzed using 50 µM LA or 50 µM GW9508 as chemoattractants.

IL-8 is a chemoattractant of neutrophils during inflammation. We demonstrated that LA increased the expression of IL-8 in HaCaT cells (**Figure 5D**). To confirm the participation of this chemokine in neutrophil chemotaxis induced by conditioned media of HaCaT cells stimulated with FFA1 agonists, we depleted IL-8 from the conditioned medium using a neutralizing antibody. We observed that depletion of IL-8 decreased migration of neutrophils into the conditioned medium of HaCaT cells stimulated with 50 µM LA and 50 µM GW9508 (**Figures 7A, B**). However, this decrease in chemotaxis was partial, and total chemotaxis was not reduced to control levels in any of the treatment conditions. Also, when LA or GW9508 were used directly as chemoattractants, no significant increase in neutrophil chemotaxis toward the culture medium was observed (**Figures 7C, D**). Therefore, these results suggested that LA-induced production and secretion of IL-8 in HaCaT cells *via* FFA1 receptor, and IL-8–mediated migration of neutrophils.

## Discussion

**Figure 8:**
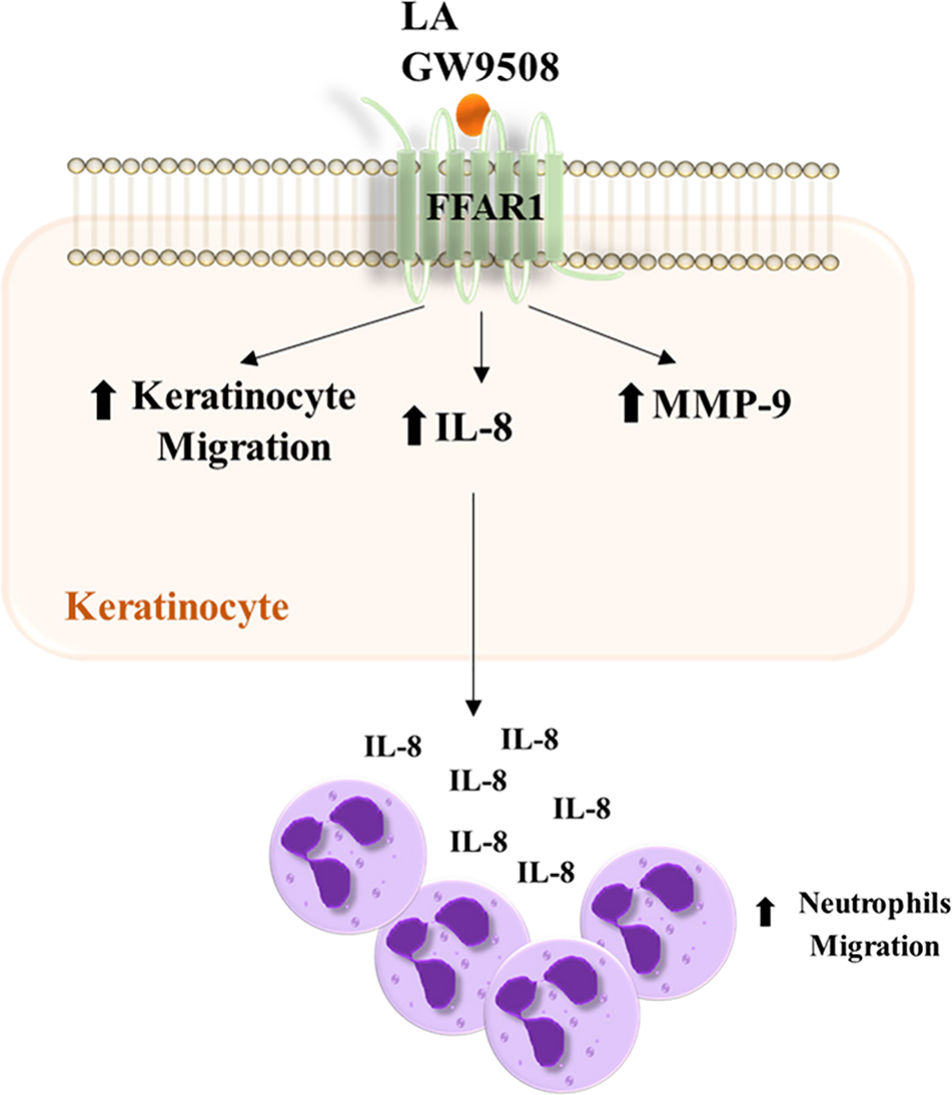
Summary scheme of the effects produced by LA and GW9508 on HaCaT cells and their role in neutrophil migration.

In this study we demonstrated that activation of FFA1 receptor signaling regulates migration, MMP-9 activity, and IL-8 expression in HaCaT cells. Furthermore, we demonstrate that conditioned media from HaCaT cells stimulated with FFA1 agonists increase neutrophil chemotaxis (**Figure 8**). We first found that LA increases the migration of HaCaT cells and the FFA1 receptor is expressed in these cells. In agreement with our results, Fujita et al. (2011) demonstrated the expression of FFA1 mRNA in HaCaT cells, but the role of FFA1 in keratinocytes and its link in wound healing is poorly understood. In the present study, the LA-induced migration of HaCaT cells was decreased by a FFA1 antagonist, GW1100. Furthermore, a synthetic agonist of FFA1 (GW9508), increased the migration of these cells.

These results suggest that stimulation of FFA1 may have a functional role on wound healing, modulating the migration of keratinocytes. Other authors have studied the effect of fatty acids/FFA1 in the migration of mesenchymal stem cells (MSC). A recent study showed that oleic acid, a monounsaturated omega-9 fatty acid, enhanced umbilical cord blood-derived mesenchymal stem cell (hUCB-MSC) motility through EphB2-dependent F-actin formation involving FFA1/PKCα/GSK3β/β-catenin and Rac1 signaling pathways (Jung et al., 2015). Similarly, arachidonic acid, an ω-6 long-chain polyunsaturated fatty acid, increased migration of hUCB-MSCs through activation of the FFA1 receptor (Oh et al., 2015).

LA and GW9508 activated the ERK1/2 and p38 signaling pathways, and when both pathways were inhibited, a decrease in LA-induced migration of HaCaT cells was observed. Our results is according with findings in human corneal epithelial cells, which showed that inhibition of ERK1/2 and p38 decreased epithelial migration induced by hepatocyte growth factor (HGF) and keratinocyte growth factor (KGF), resulting in delayed wound healing in an *in vitro* assay (Sharma et al., 2003). We observed that GW1100 decreased LA-induced phosphorylation of ERK1/2 and p38. These results suggest that the stimulation of FFA1 by LA increases the phosphorylation of ERK 1/2 and p38, resulting in migration of HaCaT cells. Our results agree with previous studies that showed an increase in the activation of ERK1/2 and p38 MAPK in HEK293 cells stably transfected with human FFA1 in response to LA treatment (Qian et al., 2017). In addition, stimulation of FFA1 induced rapid activation of p38 MAPK in osteocytes and bovine neutrophils (Mieczkowska et al., 2012; Mena et al., 2016).

ERK1/2 and p38 regulate the expression of MMP-9 through the activation of the transcription factor AP-1 (activator protein-1) in keratinocytes treated with TNFα. MMP-9 (92 kDa) is the main gelatinase responsible for degradation of collagen IV to facilitate migration of basal keratinocytes during wound healing (Raja et al., 2007). Our results showed that LA and GW9508 increased MMP-9 activity, and these results were consistent with the effects of both agonists on migration of HaCaT cells. Previous reports have shown that LA and GW9508 increased the release of MMP-9 from granules stored in bovine neutrophils (Mena et al., 2013; Manosalva et al., 2015). In addition, another study showed that LA increased the expression and activity of MMP-9 in breast cancer cell lines, which was associated with increased migration of these cells (Gonzalez-Reyes et al., 2018).

A previous study showed that LA accelerated the early inflammatory phase, resulting in increased numbers of neutrophils in the wound (Rodrigues et al., 2012). Keratinocytes are important producers of chemotactic cytokines including chemokine ligand 1 (CXCL-1), IL-8, IL-1β, and interleukin-6 (IL-6), which were identified as the main chemotactic cytokines in fluids in human wounds (Wertheimer et al., 2001; Lundahl et al., 2012). We demonstrated that LA and GW9508 increased the expression of IL-8 in HaCaT cells, which was reduced by treatment with GW1100. IL-8 is an important chemoattractant which modulates CD11b in neutrophils to promote extravasation (Lundahl et al., 2012). The conditioned media of HaCaT cells treated with LA or GW9508 increased chemotaxis of neutrophils, which was inhibited by neutralization of IL-8 using an anti-IL-8 antibody. These results suggested that FFA1 agonists induced the expression of IL-8 in HaCaT cells, resulting in IL-8 secretion into the media to promote migration of neutrophils. When LA or GW9508 were used directly as chemoattractants, no significant increase in neutrophil chemotaxis toward the culture medium was observed. These results are in accordance with the fact that human neutrophils do not express the FFA1 receptor (Hidalgo et al., 2011; Manosalva et al., 2015). In addition, previous studies have shown that FFA1 agonists directly increased migration of bovine neutrophils (Mena et al., 2013), which express the receptor (Hidalgo et al., 2011; Manosalva et al., 2015). Therefore, our results suggested that keratinocytes can modulate the early inflammatory response by paracrine stimulation of neutrophils through secretion of chemokines, such as IL-8 which can be induced by FFA1 agonist. Evidence suggests that altered recruitment of neutrophils can disrupt wound healing kinetics, by delaying the passage from inflammatory phase to the proliferative phase (Guo and Dipietro, 2010; Ebaid, 2014; Makrantonaki et al., 2017). As observed in our study, linoleic acid increases the production of IL-8 in keratinocytes favoring the migration of neutrophils, which has an essential function in the inflammatory phase of the healing process. Hence, this suggests that linoleic acid and agonists of FFA1 can accelerate the onset of the following stages (reepithelialization) and thus accelerate the entire healing process. Therefore, restore synchronization of neutrophil recruitment as well as its function, can be a treatment goal to linoleic acid y FFA1 agonists to promote wound healing.

Finally, this study demonstrates that linoleic acid and FFA1 agonists can modulate the cellular response, increasing the migration of keratinocytes and inflammatory response, suggesting that FFA1 may be a pharmacological target for the treatment of wounds. However, the partial effect of the antagonist suggests that other mechanisms could be involved in the HaCaT cells responses induced by LA.

## Conclusions

In conclusion, our findings demonstrated that linoleic acid through FFA1 increased migration of HaCaT cells by activating of the ERK1/2 and p38 signaling pathway. In addition, increased MMP-9 activity induced by linoleic acid may contribute to this process. Furthermore, we observed that keratinocytes stimulated with LA increased expression of IL-8, which may have contributed to increased neutrophil chemotaxis. Therefore, the present study contributes to the understanding of the mechanisms by which linoleic acid could favor wound healing through modulation of FFA1 in keratinocytes.

## Data Availability Statement

All datasets generated for this study are included in the article/**Supplementary Materia**l.

## Ethics Statement

The studies involving human participants were reviewed and approved by the ethical committee of the Universidad Austral de Chile. The patients/participants provided their written informed consent to participate in this study.

## Author Contributions

CM designed the experiments. PA, KG, KI, JS, and FP performed the experiments. CM, PA, MH, GM, and RB contributed to the data analysis and writing of the manuscript. All authors reviewed the manuscript.

## Funding

This work was supported by the grants from Universidad Austral de Chile, DID-UACH S-2014-13 and S-2016-07. Fondecyt Post-doctorado (3170775) and Fondecyt (1151047).

## Conflict of Interest

The authors declare that the research was conducted in the absence of any commercial or financial relationships that could be construed as a potential conflict of interest.

## Supplementary Material

Supplementary Figure 1 Assay of transwells of HaCaT cells, determination of% stained area. Stained area of HaCaT cells stimulated with **(A)** 50–100 mM LA; **(B)** HaCaT cells pre-incubated with GW1100 and stimulated with 50 mM LA; **(C)** 10–50 mM GW9508; **(D)** pre-incubated HaCaT cells 10 mM U0126 or 10 mM SB203580 and stimulated with 50 mM LA.
