# Supplementary figures and images for: Free Fatty Acid Receptor 1 Signaling Contributes to Migration, MMP-9 Activity, and Expression of IL-8 Induced by Linoleic Acid in HaCaT Cells
Source: Front Pharmacol. 2020 May 5;11:595. doi: 10.3389/fphar.2020.00595 (PMC7216565; doi:10.3389/fphar.2020.00595)

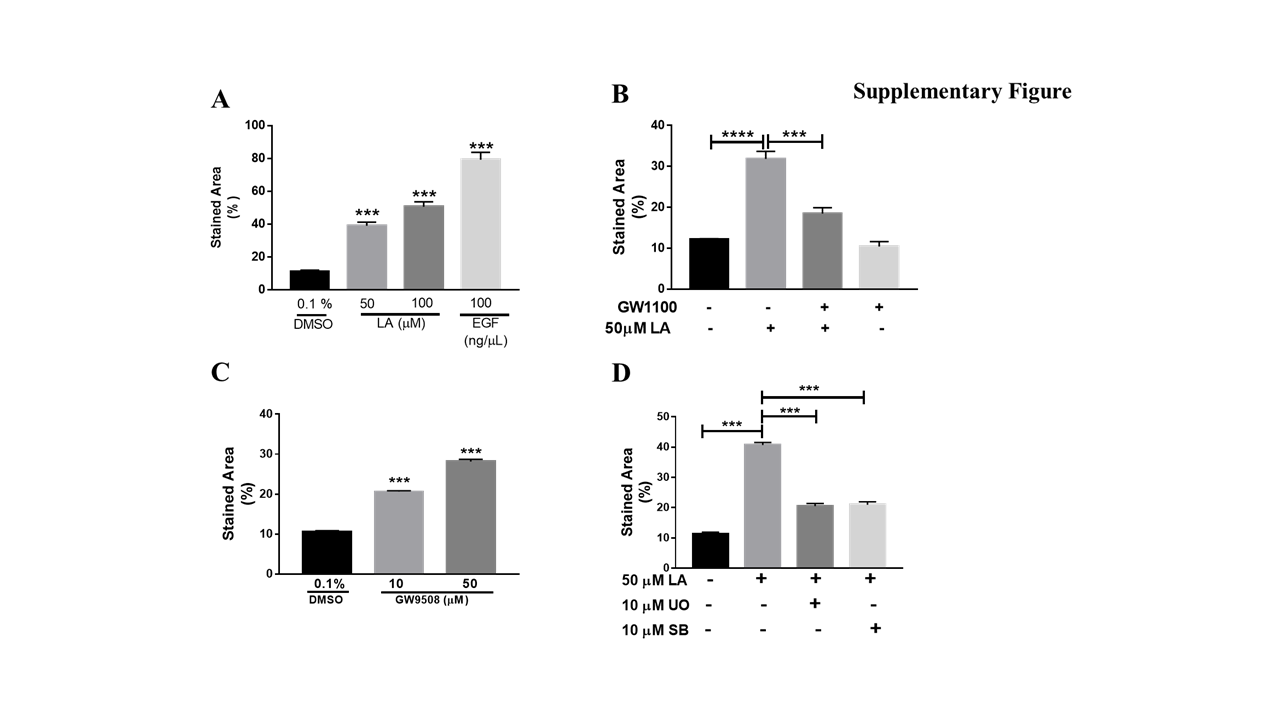

Supplement: Supplementary Figure 1 — Assay of transwells of HaCaT cells, determination of% stained area. Stained area of HaCaT cells stimulated with (A) 50–100 mM LA; (B) HaCaT cells pre-incubated with GW1100 and stimulated with 50 mM LA; (C) 10–50 mM GW9508; (D) pre-incubated HaCaT cells 10 mM U0126 or 10 mM SB203580 and stimulated with 50 mM LA. [file Image_1.tif]
